
JOURNAL INFORMATION
==============================
NLM Title Abbreviation: ScientificWorldJournal
Journal ID: ScientificWorldJournal
NLM Journal ID: 101131163
Journal ID: TSWJ

The Scientific World Journal

ISSN: 2356-6140
EISSN: 1537-744X
Publisher: Wiley

ARTICLE INFORMATION
==============================
PMCID: PMC6009752
PMID: 29973806
DOI: 10.1100/tsw.2002.32
Article ID: 829135
Article version: 1
Subjects: Short Report

Structure and Mechanism of E. coli Rnase

Zuo Yuhong
Deutscher Murray P.
Department of Biochemistry and Molecular Biology, University of Miami School of Medicine, P.O. Box 016129, Miami, FL 33101, USA
Publication date: 2002
Electronic publication date: 2002 May 4
Volume: 2
First page: 65
Last page: 66
Copyright: Copyright © 2002 Yuhong Zuo and Murray P. Deutscher.
Copyright year: 2002
License: This is an open access article distributed under the Creative Commons Attribution License, which permits unrestricted use, distribution, and reproduction in any medium, provided the original work is properly cited.
License URL: https://creativecommons.org/licenses/by/3.0/

==============================
Advances in Gene Technology: The Genome and Beyond –
Structural Biology for Medicine (Proceedings of the 2002 Miami
*Nature Biotechnology* Winter Symposium)
*TheScientificWorld* 2002, 2(S2), 65–66
ISSN 1532-2246; DOI 10.1100/tsw.2002.32

### STRUCTURE AND MECHANISM OF *E. coli* RNASE T

Yuhong Zuo and Murray P. Deutscher
Department of Biochemistry and Molecular Biology, University of Miami School of Medicine,
P.O. Box 016129, Miami, FL 33101

**INTRODUCTION.** RNase T is one of eight distinct 3’ to 5’ exoribonucleases identified in *Escherichia coli*[1]. It belongs to the DEDD exonuclease superfamily[2] characterized by common motifs consisting of four invariant acidic residues, which in DNA polymerases were shown to form the exonuclease active site[3]. RNase T plays an important role in stable RNA metabolism, including tRNA end turnover and 3’ maturation of many stable RNAs[1]. RNase T proteins are small polypeptides of about 220 amino acids. They are closely related to the proof-reading domains/subunits of bacterial DNA polymerase III, and interestingly, *E. coli* RNase T also displays strong DNA exonuclease activity[4,5]. *E. coli* RNase T must form a homodimer in order to function[1].

**METHODS.** Site-directed mutageneses were PCR-based using synthetic oligonucleotides. RNase T activity against tRNA was measured by an acid-soluble assay, whereas RNase T processing of oligonucleotides was analyzed by denaturing polyacrylamide gel electrophoresis. The NCBI nonredundant protein and nucleic acid databases were searched by **BLAST** programs for RNase T orthologs. Multiple sequence alignment was generated by ClustalX with some editing based on BLAST searches and predicted secondary structures, which were predicted by **3D-PSSM** and **PredictProtein**. Homology modeling was carried out by **Modeller4** installed on PC Linux system.

**RESULTS.** To investigate the catalytic mechanism of RNase T and its requirement for dimerization, we carried out a systematic mutagenic study. Based on this study, the conserved acidic residues along with some other residues of the three DEDD exonuclease motifs were shown to be important for RNase T catalytic activity, consistent with a possible common catalytic mechanism of all DEDD members. In addition, we also identified in RNase T orthologs three other conserved sequence segments that contain a high level of positively-charged and aromatic residues. Structural modeling by threading the RNase T sequence through the *E. coli* exonuclease I crystal structure (PDB: **1FXX**) indicates that these three segments cluster together to form a putative nucleic acid-binding patch, which locates on the opposite side of the DEDD catalytic center in the RNase T monomer. Kinetic analyses of the corresponding mutants confirmed the involvement of this positively-charged/aromatic patch in nucleic acid-binding. By complementing two inactive RNase T mutants which lost either catalytic activity or nucleic-acid binding capability, RNase T activity could be partially reconstituted (Fig. 1). This result supports a structural model in which the catalytic center is derived from one subunit and the major substratebinding site comes from the other subunit, and it provides an explanation for the dimerization requirement of *E. coli* RNase T.

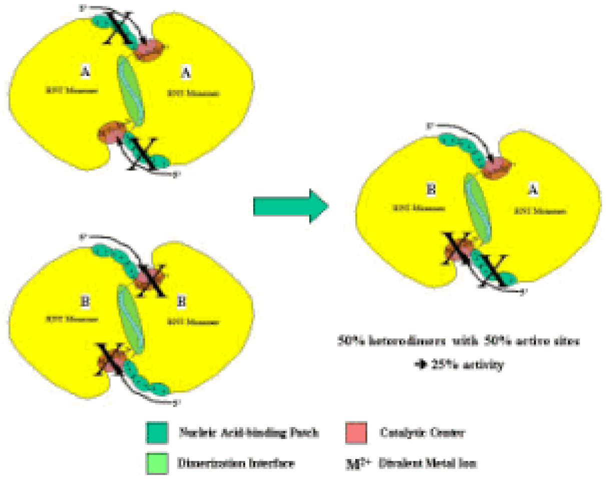

**Fig.1 Reconstitution of RNase T activity from two inactive mutants**

### REFERENCES

1. Deutscher, M.P. and Li, Z. (2000) Prog. Nucl. Acid Res. Mol. Biol. 66, 67–105.
2. Zuo, Y. and Deutscher, M.P. (2001) Nucl. Acids Res. 29(5), 1017–1026.
3. Steitz, T.A. and Steitz, J.A. (1993) Proc. Natl. Acad. Sci. U. S. A. 90(14), 6498–6502.
4. Viswanathan, M., Dower, K.W., and Lovett, S.T. (1998) J. Biol. Chem. 273(52), 35126–35131.
5. Zuo, Y. and Deutscher, M.P. (1999) Nucl. Acids Res. 27(20), 4077–4082.
